# Use of Reflectance Confocal Microscopy for Hidrocystomas: An Emerging, Cost-Effective, and Powerful Tool

**DOI:** 10.1155/2021/5543803

**Published:** 2021-04-09

**Authors:** Amanda Walker, Vikram Nath Sahni, Dev Ram Sahni, Julia Curtis

**Affiliations:** ^1^University of Utah, School of Medicine, Salt Lake City, UT 84132, USA; ^2^Drexel University, College of Medicine, Philadelphia, PA 19129, USA; ^3^University of Utah School of Medicine, Department of Dermatology, Salt Lake City, UT 84132, USA

## Abstract

Reflectance confocal microscopy (RCM) is an emerging and noninvasive imaging tool in dermatological practice. Benefits of this modality include differentiation between benign and malignant skin lesions, prevention of unnecessary biopsies, and cost effectiveness. However, RCM findings for benign lesions are rarely reported in the literature. We describe a case of reflectance confocal microscopy findings of a hidrocystoma and review potential applications of this imaging technique in everyday clinical practice.

## 1. Introduction

In vivo reflectance confocal microscopy (RCM) is a powerful and noninvasive imaging tool. RCM can be utilized in conjunction with dermoscopy to differentiate between malignant and benign cutaneous lesions. However, because most benign lesions are rarely imaged, the differentiation between malignant and benign skin lesions such as hidrocystomas when using RCM can be difficult.

Hidrocystomas are benign cystic tumors composed of eccrine or apocrine sweat glands [1, 2]. Eccrine hidrocystomas are located in the periorbital and malar regions and have a female predilection. Apocrine hidrocystomas present on the head, neck, and eyelid margin and occur equally in both genders [1, 2]. Hidrocystomas typically present as dome-shaped papules. Eccrine hidrocystomas are flesh-colored to light blue and smaller in size (1–6 mm), whereas apocrine hidrocystomas are dark blue to black and larger in size (3–15 mm) [1, 2]. Although these lesions are benign clinically, they are often mistaken for basal cell carcinoma, syringomas, milia, and blue nevi [1, 2]. Additionally, hidrocystomas can sometimes share similar dermoscopic features with basal cell carcinoma such as a blue-gray hue, making diagnosis without biopsy difficult [3, 4].

 Through utilization of RCM, providers can detect smaller malignant neoplasms and avoid unnecessary painful and scarring biopsies, as well as prevent further morbidity from missed neoplastic diagnoses [5]. We describe a case of RCM findings of a hidrocystoma and explain the utility and application of this tool in clinical practice.

## 2. History of the Present Illness

**Figure 1:**
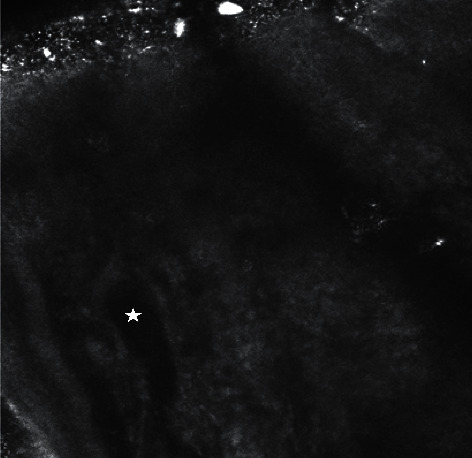
Homogenous tubular cyst-like structure (indicated by the star). The CaliberID 3000 handheld device was utilized. The depth of this image was taken at 60 *μ*m.

**Figure 2:**
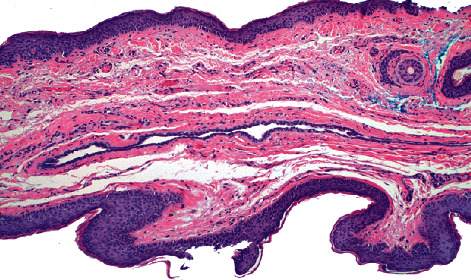
A cystic space lined by two layers of epithelial cells. Decapitation secretion can also be seen in the cyst-like space (100x H and E).

A patient presented to the clinic for a skin check. On exam, there was a 3–4 mm grey shiny papule on the left medial lower eyelid. Dermoscopic exam revealed few vessels and a grey hue. RCM revealed a homogenous tubular cyst-like structure (Figure 1). Cutaneous biopsy revealed a cystic structure lined by a double layer of epithelial cells with decapitation secretion, confirming the diagnosis of hidrocystoma (Figure 2).

## 3. Discussion

Reflectance confocal microscopy can be a cost-effective, sensitive, and specific tool and is gaining popularity in ophthalmological and dermatological practice. RCM is more sensitive than slit-lamp examination for identification of basal cell carcinoma involving the eyelid margin and conjunctiva [6]. Furthermore, reflectance confocal microscopy was in concordance with histopathologic findings in 48 of 49 BCCs, demonstrating a sensitivity of 98% for the diagnosis of basal cell carcinoma [6]. While RCM findings for BCC are well known in the literature, those for hidrocystomas are severely lacking. RCM features of hidrocystomas include homogenous cystic structures adjacent to normal‐appearing adnexal structures lacking tumor islands or silhouettes [2]. The most common clinical differential diagnosis for hidrocystomas includes basal cell carcinoma, syringomas, blue nevi, and milia. While clinical similarity can make the diagnosis challenging, RCM can aid in differentiation between hidrocystomas and these entities. Characteristic RCM findings for BCC include elongated cells with polarized nuclei intermixed with inflammatory cells, dark silhouettes, bright tumor islands, and abundant vasculature and pleomorphism of the overlying epidermis [7, 8]. RCM findings for syringomas include round to oval highly refractive and relatively monomorphous masses with dark line structures correlating to the ductal lumina [9]. Milia demonstrate refractive global keratin in the superficial dermis [9]. Blue nevi exhibit hyperreflective cells with plump cellular bodies and long dendrites intermingled with elongated collagen fibers [10].

The clinical benefits of utilizing RCM include prevention of unnecessary biopsies, decreased pain, improved cosmetic outcomes, and enhanced surveillance for recurrent malignancy [5]. Systematic use of RCM in a hospital setting reduced over 70% of the excisions of benign nevi vs. melanoma [5]. Assuming a stable number of diagnoses of melanoma over one year, this equates to a total of 3,053 unnecessary excisions that could be avoided. RCM also reduced the number needed to excise (NNE) from 19.41 with dermoscopy alone to 6.25 in combination with RCM [5]. Projected cost savings of 260,000 euros were attributed to prevention of unnecessary biopsies [5]. RCM and clinical exam can diagnose melanoma together in a single visit and result in excision concurrently, which improves patient outcomes from those lost to follow-up [11].

RCM imaging of the skin was granted codes for reimbursement by the US Centers for Medicare and Medicaid Service in 2016 and may be utilized as a cost-effective tool in dermatology [12]. However, additional imaging and training in the identification of benign and malignant lesions needs to occur. We provide further evidence for the utility of RCM in clinical practice and report a case of RCM findings of a hidrocystoma.
